# Acute Pancreatitis Associated With Hypothermia: An Uncommon Presentation

**DOI:** 10.7759/cureus.8859

**Published:** 2020-06-27

**Authors:** Muhammad Z Khan, Hamza Yousaf, Abdur Jamil, Muhammad Shah zaib, Nicholas Haddad

**Affiliations:** 1 Internal Medicine, Central Michigan University College of Medicine, Saginaw, USA; 2 Internal Medicine, Nishtar Medical University and Hospital, Multan, PAK; 3 Internal Medicine, Central Michigan University, Saginaw, USA; 4 Infectious Disease/internal Medicine, Central Michigan University College of Medicine, Saginaw, USA

**Keywords:** acute pancreatitis, hypothermia, external rewarming, mrcp, ercp, ct, us, bair hugger

## Abstract

Hypothermia is not known as a common cause of acute pancreatitis, although there have been previously reported cases. Herein, we describe a 55-year-old man who presented with acute pancreatitis preceded by hypothermia in the absence of the more traditional causative factors, such as gallstones or alcohol consumption. On arrival to the emergency department, he was found to have a temperature of 84.3°F, consistent with moderate hypothermia, a blood pressure of 84/50 mmHg, and a heart rate of 60 bpm. Serum lipase and amylase levels were 2,225 and 980 U/L, respectively. A CT scan of the abdomen with intravenous (IV) contrast revealed peripancreatic fat stranding, consistent with the diagnosis. No evidence of gallstones or common bile duct dilatation was found. He had also developed acute kidney injury and lactic acidosis consistent with end-organ damage. After appropriate triage, he was admitted to the intensive care unit and supportively managed. An external rewarming strategy with IV fluids and antibiotics resulted in improvement in clinical status. Hypothermia can cause subtle changes in the microvasculature and production of free radicals, which can result in acute pancreatitis. It is important to determine the etiology so that appropriate treatment can be instituted with better outcomes.

## Introduction

Acute pancreatitis is defined by the revised Atlanta criteria by two of the following three features: characteristic epigastric pain radiating to the back, serum amylase or lipase three times the upper limit of normal, and imaging findings on the CT scan [1]. The incidence approaches to about 5-80 cases per 100,000 [2]. Gallstones and alcohol account for majority of the cases. Although a few cases have been reported in the literature regarding hypothermia-related pancreatitis, a definite causal association has not been clearly defined [3]. It is imperative though to recognize the etiology promptly so that appropriate treatment can be instituted. Our case highlights this important concept and outlines the management plan. Hypothermia-induced pancreatitis is a rare entity. Clinicians need to be cognizant about it and should take all the necessary steps for excluding the other common causes before reaching a diagnosis.

## Case presentation

**Figure 1:**
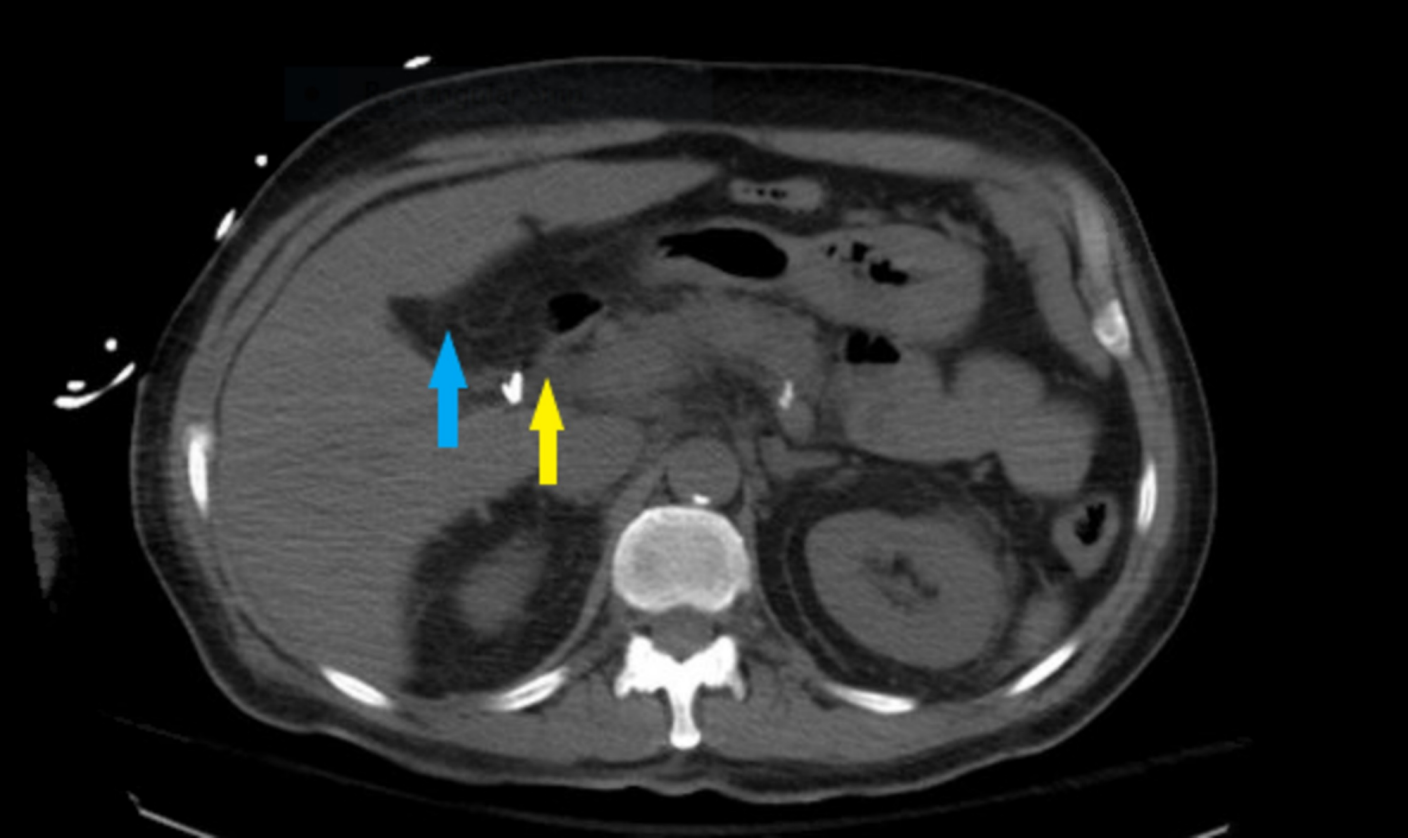
CT scan of the patient with intravenous contrast. CT scan reveals peripancreatic fat  stranding (yellow arrow). The absence of the gallbladder can be noted in the gallbladder fossa as the patient had a cholecystectomy in the past (blue arrow).

We describe the case of a 55-year-old diabetic man with a history of cholecystectomy who presented with acute pancreatitis in the setting of hypothermia. The patient had no previous history of alcohol abuse. On arrival to the emergency department, he was found to have a temperature of 84.3°F, consistent with moderate hypothermia, a blood pressure of 84/50 mmHg, and a heart rate of 60 bpm. He reported radiating epigastric pain with abdominal tenderness on physical exam and decreased consciousness. Serum lipase and amylase levels were 2,225 and 980 U/L, respectively. Blood alcohol level was undetectable. A CT scan of the abdomen with intravenous (IV) contrast revealed peripancreatic fat stranding consistent with the diagnosis (Figure 1). Hypotension had resulted in lactic acidosis and end-organ damage in the form of acute kidney injury.

Based on the clinical assessment and laboratory markers, he was admitted to the intensive care unit (ICU). After the administration of IV fluid with lactated Ringer’s solution, his blood pressure increased. Ceftriaxone and Flagyl were started initially but discontinued later when the pancultures came to be negative. Active external rewarming with a Bair Hugger convective temperature management system was initiated, and the gradual increase in body temperature resulted in improvement in his condition and biochemical markers. The lipase and amylase decreased to 904 and 400 U/L, respectively. The abdominal pain decreased in intensity, and oral feeding was resumed as tolerated. He was subsequently transferred to a stepdown unit after staying in the ICU for two days and was later discharged.

## Discussion

Acute pancreatitis is an inflammatory disorder of the pancreas with increasing incidence worldwide. Numerous etiologies have been proposed in the literature. The traditional causative factors were not associated with the case of acute pancreatitis that we encountered, which was preceded by hypothermia. Whether hypothermia can be listed as an etiology or an associated factor requires further elucidation by conducting additional research. Foulis observed microscopic or macroscopic changes in acute pancreatitis in the necropsy specimens from eight hypothermic patients [4]. Another study conducted by Maclean et al. revealed milder forms of acute pancreatitis in 53 hypothermic patients [5]. Biochemical markers indicative of acute pancreatitis are found in 50% to 65% of patients with hypothermia and 20% to 30% of necropsy findings [4,5]. Case reports can be found that further substantiate the association, such as the development of acute pancreatitis preceded by hypothermia in a 65-year-old man [6]. Postmortem analyses of 43 patients with acute pancreatitis between 1980 and 1985 included hypothermia as one of the causes [7].

The literature can be used as a platform for conducting further research to better explain the association or establish causality. Various mechanisms have been proposed for hypothermia-induced pancreatitis. Hypothermia can cause alteration of the microvasculature and generation of free radicals, which can result in an ischemic insult [4]. Savides and Hoffbrand proposed that thrombosis of the microvasculature and disseminated intravascular coagulation can result from hypothermia, which causes acute pancreatitis [8].

Diagnosis of acute pancreatitis is usually based on clinical examination and biochemical findings. Ultrasonography is considered a valuable tool to exclude gallstone-related pathology. Doppler ultrasonography has emerged as an important modality for the detection of peripancreatic fluid [9]. CT imaging provides additional detailed information regarding the anatomy and complications of pancreatitis.

It has been shown that the severity of pancreatitis can be assessed by findings on a CT scan [10]. Magnetic resonance cholangiopancreatography is another tool that has a diagnostic role in delineating biliary tract anatomy and biliary stones, but the lack of cost-effectiveness is a limiting factor. Alternatively, endoscopic retrograde cholangiopancreatography is a treatment strategy that can be readily implemented [11].

Management of the condition should be targeted at gradual external rewarming in cases of mild to moderate hypothermia. It has been shown that internal rewarming strategies do not provide any extra benefits, and complications related to coagulation and bleeding can result [12]. Uncomplicated mild to moderate acute pancreatitis in hypothermic patients is conservatively managed with efforts to normalize temperature, aggressive fluid resuscitation, and antibiotic therapy [13]. However, moderately severe or severe acute pancreatitis is managed in the ICU with consideration given to surgical therapy based on the complications. Oral feeding should be initiated as soon as tolerated, as it has been shown to reduce mortality, morbidity, and hospital stay [14]. In severe pancreatitis, enteral feeding by nasogastric tube or gastroduodenal route is preferred over parenteral nutrition, as the latter causes numerous complications and increases morbidity and mortality.

## Conclusions

Acute pancreatitis is very common in Western countries, although it is rarely encountered in association with hypothermia. Understanding the pathophysiology of acute pancreatitis in the context of hypothermia is important, and there is room for further studies that will elucidate the process. A review of the literature indicates that there is a subtle association between the two. Better outcomes are achieved if appropriate management is instituted, as evident from the case discussion.
